# Aryl Hydrocarbon Receptor (AhR) Signaling in Colonic Cells and Tumors

**DOI:** 10.3390/receptors2010005

**Published:** 2023-02-08

**Authors:** Stephen Safe, Huajun Han, Arul Jayaraman, Laurie A. Davidson, Clinton D. Allred, Ivan Ivanov, Yongjian Yang, James J. Cai, Robert S. Chapkin

**Affiliations:** 1 Department of Veterinary Physiology and Pharmacology, Texas A&M University, College Station, TX 77843, USA; 2 Program in Integrative Nutrition and Complex Diseases, Department of Nutrition, Texas A&M University, College Station, TX 77843, USA; 3 Department of Chemical Engineering, Texas A&M University, College Station, TX 77843, USA; 4 Department of Nutrition, University of North Carolina at Greensboro, Greensboro, NC 27412, USA; 5 Department of Veterinary Integrative Biosciences, Texas A&M University, College Station, TX 77843, USA

**Keywords:** Ah Receptor, Colon, Stem Cells, FOXM1

## Abstract

The aryl hydrocarbon receptor (AhR) is overexpressed in many tumor types and exhibits tumor-specific tumor promoter and tumor suppressor-like activity. In colon cancer, most but not all studies suggest that the AhR exhibits tumor suppressor activity which is enhanced by AhR ligands acting as agonists. Our studies investigated the role of the AhR in colon tumorigenesis using wild-type and AhR-knockout mice, the inflammation model of colon tumorigenesis using mice treated with azoxymethane (AOM)/dextran sodium sulfate (DSS) and APC^S580/+^; Kras^G12D/+^ mice all of which form intestinal tumors. The effects of tissue-specific AhR loss in the intestine of the tumor-forming mice on colonic stem cells, organoid-initiating capacity, colon tumor formation and mechanisms of AhR-mediated effects were investigated. Loss of AhR enhanced stem cell and tumor growth and in the AOM/DSS model AhR-dependent suppression of FOXM1 and downstream genes was important for AhR-dependent anticancer activity. Furthermore, the effectiveness of interleukin-22 (IL22) in colonic epithelial cells was also dependent on AhR expression. IL22 induced phosphorylation of STAT3, inhibited colonic organoid growth, promoted colonic cell proliferation in vivo and enhanced DNA repair in AOM/DSS-induced tumors. In this mouse model, the AhR suppressed SOCS3 expression and enhanced IL22-mediated activation of STAT3, whereas the loss of the AhR increased levels of SOCS3 which in turn inhibited IL22-induced STAT3 activation. In the APC^S580/+^; Kras^G12D/+^ mouse model, the loss of the AhR enhanced Wnt signaling and colon carcinogenesis. Results in both mouse models of colon carcinogenesis were complemented by single cell transcriptomics on colonic intestinal crypts which also showed that AhR deletion promoted expression of FOXM1-regulated genes in multiple colonic cell subtypes. These results support the role of the AhR as a tumor suppressor-like gene in the colon.

## 1. Introduction:

The aryl hydrocarbon receptor (AhR) is a basic helix-loop-helix protein that was initially discovered as the intracellular receptor that bound the environmental toxicant 2,3,7,8-tetrachlorodibenzo-p-dioxin (TCDD, dioxin) with high affinity [1]. Subsequent studies showed that TCDD and structurally related chlorinated dibenzo-p-dioxins (PCDDs), dibenzofurans (PCDFs) and biphenyls (PCBs) also bound the AhR and there was a correlation between their receptor binding affinities and their toxic and biochemical potencies in cellular and animal models [2]. TCDD and related halogenated aromatics induced a common pattern of age, sex and species-dependent toxic responses including a wasting syndrome, chloracne, hepatic porphyria, thymic atrophy and teratogenicity [2]. The classical mechanism of action for dioxin-like compounds (DLCs) involves ligand binding to the cytosolic AhR, nuclear translocation and formation of a heterodimer with the AhR nuclear translocator (ARNT) protein and activation of gene expression through binding of the heterodimer to cis-acting AhR response elements (AhREs) in target gene promoters [3,4].

**Figure 1.**
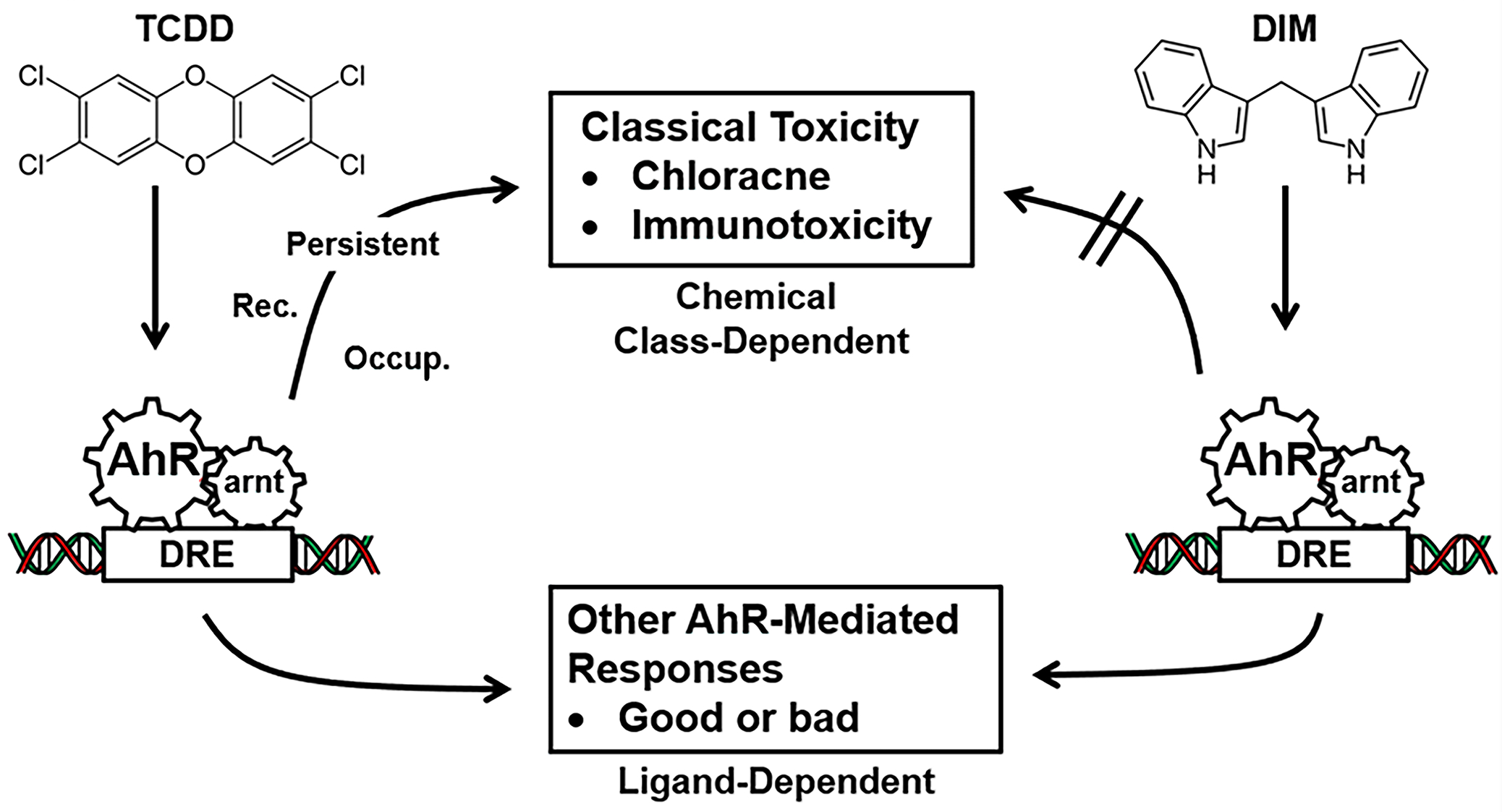
AhR ligands as Selective AhR Modulators (SAhRMs). The toxicities associated with TCDD, and related compounds is associated with their persistence and as yet other unknown factors [7–9] whereas SAhRMs such as diindolylmethane (DIM) do not induce the toxic responses.

A number of subsequent studies demonstrated that the AhR was not only a receptor that mediated the toxic effects of a specific small set of structurally related toxicants but also had multiple endogenous functions for maintaining cellular homeostasis and patho-physiology [5,6]. It was also discovered that the AhR also bound and is activated by structurally diverse ligands including health promoting phytochemicals, microbial metabolites, endogenous biochemicals, pharmaceuticals and many other structurally diverse compounds [7–9]. Development of AhR knockout mice demonstrated that the AhR has multiple functions in organs/tissues [10–14]. Differences in the effects of AhR ligands are due, in part, to their activity as selective AhR modulators (SAhRMs) and the tissue persistence of the toxic AhR ligands (Figure 1).

The AhR has emerged as a potential drug target for multiple diseases including cancer; however, there are still some conflicting reports regarding the pro-oncogenic or tumor suppressor-like activity of the receptor and its ligands and this is particularly true for breast cancer [15,16]. Some reports show that the AhR exhibits pro-oncogenic activity in colon cancer [17–19]; however, most studies on colon cancer indicate that the AhR is a tumor suppressor and tumor growth is inhibited by AhR agonists [20–23]. This paper will highlight the role of the AhR in colon cancer and the mechanisms associated with its anticancer activities.

## 2. AhR and Its Role in Colon Cancer

The role of the AhR in colonic inflammation models of inflammatory bowel disease and colon cancer have been previously investigated in cell culture and in in vivo models and with some exceptions, the AhR and selected agonists have been associated with decreased colonic inflammation and increased tumor suppression [20,21]. AhR^−/−^ knockout mice develop cecal tumors with high accumulation of beta-catenin in the tumors whereas this response was not observed in heterozygous or wild type mice (20). Cecal carcinogenesis was also observed in AhR^−/−^ and AhR^+/−^ and AhR^+/+^ mice crossed with the Apc^min/+^ mouse with a decreasing order of susceptibility, respectively, and cecal tumorigenesis was inhibited in AhR-expressing mice or after dietary treatment with AhR ligands indole-3-carbinol and diindolylmethane [0.1 and 0.01%, respectively, in the diet]. Cecal tumor formation is also enhanced by microbial bacteria and apoptosis-associated speck such as proteins containing a caspase recruitment domain (ASC) [21]. The AhR and AhR agonists also inhibited colon tumor formation in an inflammation model of colon cancer where mice are treated with the carcinogen azoxymethane (AOM) in combination with inflammatory stressor dextran sodium sulfate (DSS), and in a syngeneic mouse model using MC38 colon cancer cells injected into the right flank [22]. In contrast, some studies reported alternative results showing that the AhR exhibited pro-oncogenic activity primarily in colon cancer cell models [17–19]. Our studies investigated the role of the AhR and AhR ligands in various models of colon carcinogenesis and also focused on the mechanisms associated with the tumor suppressor-like activity of this receptor.

## 3. Inflammation-Associated Colon Carcinogenesis-Role of AhR

**Figure 2.**
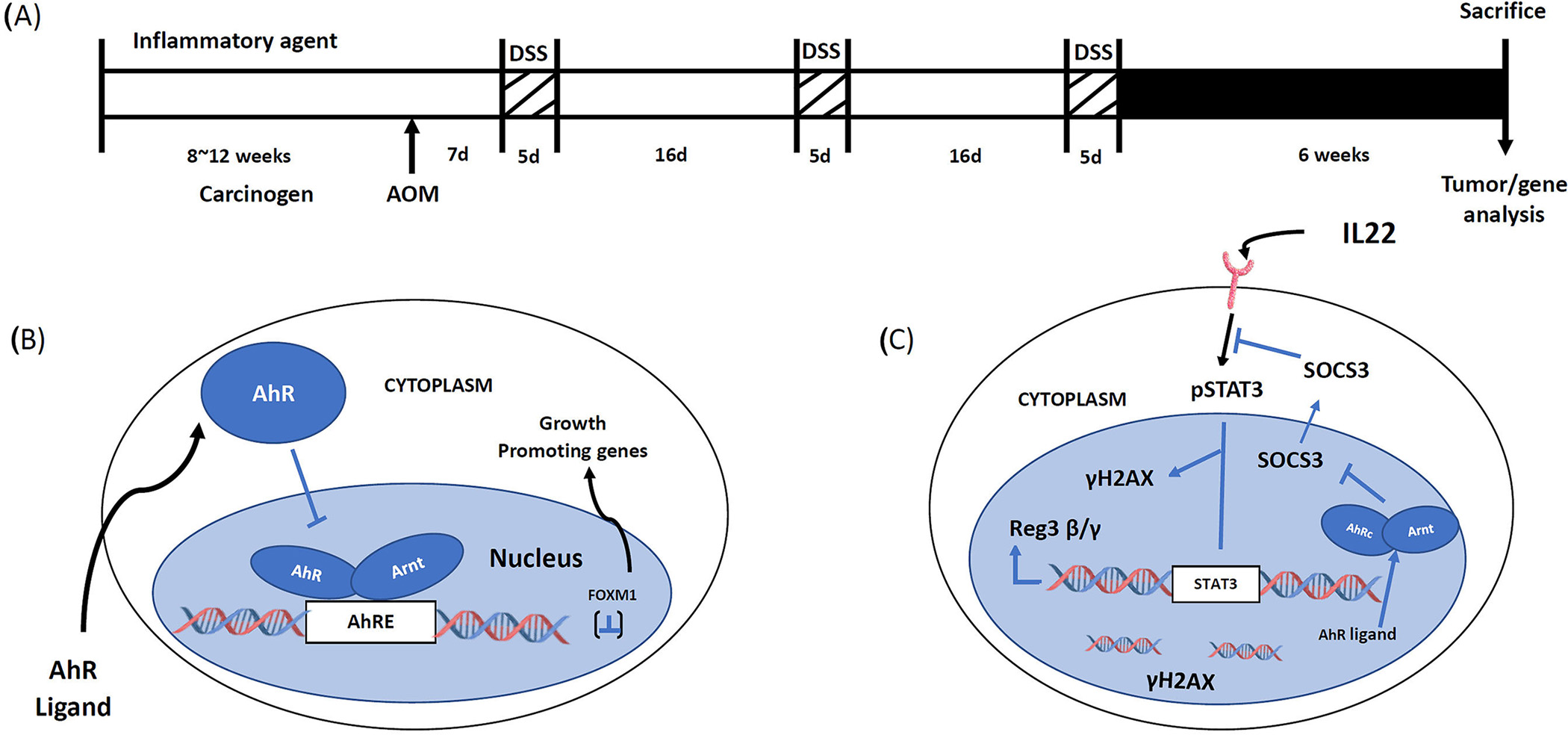
Experimental protocol and mechanism of action of the AhR in colon cancer (**A**). Model for the AOM/DSS experimental protocol [25] (**B**). The AhR inhibits growth of colon tumors by suppressing expression of FOXM1 [25] (**C**). The AhR enhances IL22-mediated activation of STAT3 and downstream pathway by suppressing expression of SOCS3 [33]. The figures represent intestinal cells and IL22 is generated by ILCs and other immune cells.

The inflammation-induced colon cancer mouse model [23–25] was used for investigating the mechanisms of AhR-mediated effects on colonic stem cells and colon tumor formation [23,24]. This model used AOM (10 mg/kg) as the carcinogen which is then promoted by three cycles of DSS followed by termination 6 weeks after the final dose of DSS (Figure 2A). A comparison of AhR^+/+^ and AhR^−/−^ mice in the combined carcinogen-inflammation (AOM/DSS) model showed that significantly higher levels of overall tumor incidence, the number of adenomas per mouse, tumor volume and the number of adenocarcinomas per mouse were observed in the AhR knockout mice [25]. These data complement results of a previous study using this same model [23]. Since colon stem cells are precursors of intestinal tumors [26], we used an inducible deletion of the AhR in an Leucine-rich-containing G-protein coupled receptor 5 (LGR5) expressing model and examined the role of the AhR in colonic stem and progenitor cells. Loss of the AhR had dramatic effects on stem and progenitor cells and these included increased organoid forming efficiency and diameter whereas some parameters were decreased by treatment with 25 nM TCDD in wild type but not AhR^−/−^ cells. The observation that the AhR and TCDD treatment decrease colonic stem and progenitor cells correlated with the AhR-dependent decrease of colonic tumor formation. RNAseq and subsequent pathway analysis of differentially expressed genes in stem and progenitor cells from AhR^+/+^ and AhR^−/−^ mice demonstrated that the AhR repressed Forkhead box protein M1(FOXM1) expression which was further decreased by TCDD. AhR-dependent repression of FOXM1 was observed in crypts adjacent to colon tumors and tumors, stem and progenitor cells and chromatin immunoprecipitation showed that TCDD induced formation of the AhR:ARNT complex in regions of the FOXM1 promoter containing a cis-acting AhRE binding site (Figure 2B). The discovery that the AhR represses FOXM1 expression in the colon is consistent with a previous report showing that FOXM1 signaling contributes to formation and growth of colonic tumors (Figure 2A) [27].

Interleukin 22 (IL22) plays an important functional role in the gastrointestinal tract by maintaining gut barrier function, protecting against inflammation, enhancing wound associated regeneration and responsiveness to DNA damage. IL22 is produced by different types of immune cells including innate lymphoid cells (ILCs) and there is evidence that the AhR plays a role in increased cellular levels of IL22 [28,29]. The AhR or its ligands play a direct role in the induction of IL22 and amelioration of colonic inflammation and intestinal stem cell distress [30–32]. Loss of the AhR in the AOM/DSS mouse model for colon carcinogenesis was used to investigate potential interactions between the AhR and IL22 and mechanisms of this interaction and effects on colon organoids (Figure 2B) [33]. IL22 enhanced STAT3 phosphorylation in organoids and increased colonic cell proliferation in vivo. Loss of the AhR also decreased IL22-responsiveness and blunts the DNA damage response after treatment with AOM. Examination of RNAseq data from our initial study [25] and based on the known IL22 signaling pathways resulted in identification of Suppressor of cytokine signaling 3 (SOCS3) as a critical differentially enhanced gene after AhR knockout (Figure 2C) [33]. Subsequent studies showed that AhR deficiency in organoids resulted in SOCS3 induction and treatment with TCDD decreased SOCS3 levels in AhR^+/+^ but not AhR^−/−^ mice and SOCS3 levels were also elevated in colonic crypts in the absence of AhR expression (33). The relationship between SOCS3 and the AhR was further investigated in AhR deficient mice in the AOM/DSS tumor model where SOCS3 levels were increased in tumors compared to uninvolved mucosa. Thus, AhR-SOCS3 interactions are inhibitory in intestinal cells and tumors resulting in enhanced pSTAT3 and downstream genes including the antimicrobial peptide Reg3^β/γ^ peptide and γH2AX (33). Previous studies have also demonstrated important AhR-SOCS3 interactions associated with plasmodium burghei infection [34], hepatotoxicity [35] and carcinogen-induced lesions [36] and in all of these three examples the AhR and AhR ligands induced SOCS3. Moreover, the mechanism of AhR-dependent SOCS3 induction involved interaction of the AhR complex with cis-acting AhREs in the SOCS3 promoter [28]. In contrast the AhR represses SOCS3 expression in intestinal-derived cells and this results in IL22-induced pSTAT3 and downstream signaling pathways. In the absence of the AhR, IL22 /STAT3 responsiveness is inhibited, and this compromises the activity of IL22 in maintaining gut health (Figure 2C).

## 4. Apc^S580/+^; Kras^G12D/+^ Mice and Colon Cancer: Role of the AhR

The Apc^S580/+^; Kras^G12D/+^ mouse contains an inactivating mutation of the tumor suppressor APC gene and an activating mutation of the Kras oncogene in the intestine which enhances colon tumorigenesis, and our study investigated the role of the AhR in this model by comparing results in the mutant mice with or without intestinal AhR expression [37]. Functional effects of loss of AhR on this genetic mouse model were similar to that observed in the inflammation induced mouse model of colon carcinogenesis as described above. Loss of intestinal AhR increased organoid-forming efficiency of stem and progenitor cells, enhanced organoid size and number, increased tumor size and the number of tumors in the distal colon per mouse and cecum weight. Moreover, analysis of RNAseq data showed that AhR loss enhanced Wnt signaling. In mice expressing the AhR, treatment with TCDD, showed increased AhR-responsiveness and decreased FOXM1 in organoids. Thus, in this genetic mouse model for colon tumorigenesis, AhR-mediated suppression of the Wnt signaling pathways are major tumor suppressor-like responses.

## 5. Single Cell Analysis of Colon Crypt Cells

Despite recent progress recognizing the importance of AhR-dependent signaling in colon cancer initiation and progression, its role in regulating colonic crypt homeostasis has been the subject of much speculation. Recently, it has been demonstrated that single cell multi-omics enable the characterization of previously unapproachable clinical phenomena, such as “deep landscapes” of cell heterogeneity that reflect the dynamics of the intestinal crypt [38]. Thus, to further assess the effects of AhR on intestinal epithelial cell–cell communication, we utilized single-cell RNA sequencing (scRNAseq) to assess tran-scriptomics at the single cell level in wild-type and intestinal-specific AhR knockout mice [39]. Consistent with bulk RNA findings [35], AhR deletion increased FOXM1 regulated genes in crypt-associated epithelial cell types and subtypes of goblet cells and crypt secretory cells. In addition, AhR deletion elevated single-cell entropy (a measure of differentiation potency or cell stemness) and RNA velocity length (a measure of the rate of cell differentiation) in noncycling and cycling Lgr5^+^ stem cells. In general, intercellular signaling crosstalk via soluble and membrane-bound factors was perturbed in AhR null colon-ocytes. For example, with respect to epidermal growth factor (EGF) pathway, increased EGF receptor (EGFR) interactions involving enterocytes were detected following AhR deletion. Collectively, these findings provide new evidence linking AhR with the modulation of putative stem cell driver genes, colonic crypt potency lineage decisions and cell–cell communication in vivo.

## 6. Summary

The AhR regulates anti-inflammatory activities in the gut and both the receptor, and its ligands protect against intestinal inflammation and development of colon cancer. Our research has demonstrated that in an inflammation model of colon cancer where mice are treated with the carcinogen AOM and DSS (AOM/DSS), tumor development is enhanced with loss of the AhR and AhR ligands inhibit tumorigenesis in AhR^+/+^ mice. The AhR inhibits growth of colonic stem cells, and this is also consistent with the tumor suppressor like activity of this receptor. The mechanism of AhR-mediated anti-tumorigenic activity in the AOM/DSS model involves suppression of the growth-promoting gene FOXM1. Additionally, in this same model which incorporates IL22 as an anti-inflammatory agent generated from group three innate lymphoid cells (ILC3s), the AhR suppressed SOCS3 expression and enhanced IL22-dependent activation of pSTAT3 and downstream genes whereas in AhR deleted mice, SOCS3 expression is enhanced and inhibits pSTAT3. We also observed that in APC^S580/+^; Kras^GD12/+^ mutant mice that loss of AhR activates colon stem cells and colon cancer and Wnt signaling. Single cell sequencing of intestinal crypts from AhR wild type and intestinal specific AhR knockout mice demonstrated how the AhR shaped differentiation potency in the mouse colon. Deletion of the AhR enhanced expression of FOXM1- and FOXM1-regulated genes in crypt-associated canonical epithelial cells, deep crypt-secretory cells and subtypes of goblet cells. The overall results clearly confirm the tumor suppressor-like activity of the AhR in the colon and demonstrate the possible clinical applications of AhR agonists for treating this disease.

## Data Availability

Data already available in a publicly accessible repository and on request.
